# Analysis of the *Cryptosporidium* spp. and *gp60* subtypes linked to human outbreaks of cryptosporidiosis in England and Wales, 2009 to 2017

**DOI:** 10.1186/s13071-019-3354-6

**Published:** 2019-03-12

**Authors:** Rachel M. Chalmers, Guy Robinson, Kristin Elwin, Richard Elson

**Affiliations:** 1 Cryptosporidium Reference Unit, Public Health Wales Microbiology and Health Protection, Singleton Hospital, Swansea, SA2 8QA UK; 2 Swansea University Medical School, Swansea University, Grove Building, Singleton Park, Swansea, SA2 8PP UK; 3 National Infection Service, Public Health England, 61, Colindale Avenue, London, UK; 4 National Institute for Health Research Health Protection Research Unit (NIHR HPRU) in Gastrointestinal Infections, Liverpool, UK

**Keywords:** *Cryptosporidium parvum*, *Cryptosporidium hominis*, Outbreak, Surveillance, *gp60*

## Abstract

**Background:**

*Cryptosporidium* spp. are important causes of gastroenteritis that can be transmitted from humans and animals. We elucidated the distribution of species and *gp60* subtypes in human outbreaks classified by transmission vehicle.

**Methods:**

We used a combined database of national outbreak surveillance and reference unit data to analyse outbreaks by setting, vehicle, season, and linkage with suspected sources.

**Results:**

A total of 178 outbreaks involving 4031 laboratory confirmed cases were identified; 82 (46%) outbreaks involved recreational waters, 74 (42%) animal contact, 4 (2%) environmental contact, 4 (2%) person-to-person spread, 3 (2%) food, 2 (1%) drinking water supplies, and 9 (5%) were of unknown source. The infecting *Cryptosporidium* sp. was identified in 131 (74%) outbreaks; 69 were *C. parvum*, 60 *C. hominis*, and in two outbreaks cases were infected with either species. Animal contact, environmental contact, and food-borne outbreaks were exclusively *C. parvum* and were mainly in first half of the year. Recreational water outbreaks were predominantly *C. hominis* and were mainly in the second half of the year. Outbreaks attributed to person-to-person spread were exclusively *C. hominis* and all occurred in October. Both *C. parvum* and *C. hominis* caused drinking waterborne outbreaks. *Gp60* subtypes were identified from patients in 48 *C. parvum* and 38 *C. hominis* outbreaks, revealing more subtypes among *C. parvum* (*n *= 14) than *C. hominis* (*n *= 7) outbreaks. *Cryptosporidium hominis* IbA10G2 predominated (30 outbreaks). Of *C. parvum* subtypes, IIaA15G2R1 predominated (17 outbreaks), followed by IIaA17G1R1 (12 outbreaks), IIaA19G1R1 (four outbreaks), and other subtypes caused three or fewer outbreaks each. Linkage between cases and suspected sources by *gp60* subtype was established in nine animal contact, three swimming pool, and one drinking water outbreak.

**Conclusions:**

The public health benefit of identifying infecting species and subtypes was twofold: (i) identifying and strengthening epidemiologic links between cases; and (ii) indicating possible exposures and sources to inform outbreak management. *Gp60* subtype refined the epidemiological investigations, but a multilocus genotyping scheme would provide further benefit. Characterisation of *Cryptosporidium* spp. and subtypes needs to shift from predominantly supporting outbreak investigations to becoming nationally systematic.

**Electronic supplementary material:**

The online version of this article (10.1186/s13071-019-3354-6) contains supplementary material, which is available to authorized users.

## Background

The gastrointestinal parasitic protozoans *Cryptosporidium* spp. are notifiable as causative agents of human infection in England and Wales [1]. Live laboratory reporting by diagnostic laboratories is used to collect case data and enable national surveillance by Public Health England (PHE). Numbers are variable and seasonal; 2990 to 5925 cases (mean 4341 cases) were reported annually in the years 2007–2016, with most cases in the late summer-early autumn [2]. Identification of large-scale outbreaks is based on active exceedance monitoring, and on syndromic surveillance (diarrhoea, vomiting) [3]. Practices for local exceedance monitoring, outbreak investigations and reporting vary [4], and outbreak surveillance is based on voluntary reporting; data are collated by PHE using the Outbreak Electronic Foodborne and Non-Foodborne Gastrointestinal Outbreak Surveillance System (eFOSS). Upon notification of an outbreak, a link to a web-based standardized surveillance form is sent to the lead investigator for completion once the outbreak investigation has ended.

*Cryptosporidium* outbreaks reflect the faecal-oral transmission, robustness, and chlorine resistance of this parasite, and have been linked to recreational waters (especially swimming pools), mains and private drinking water supplies, institutions such as hospitals and children’s day-care centres, food consumption, animal contact, and various environmental exposures [5]. Outbreak investigations are hampered somewhat as the incubation period is usually 5 to 7 days but can be up to 2 weeks, and by the time an outbreak is recognised recall is difficult and sampling suspected sources may not be possible or helpful. The only standard methods are for testing water [6, 7], leafy green vegetables and soft berry fruits [8].

Routine laboratory testing identifies the genus *Cryptosporidium*; species differentiation is a specialist or reference laboratory test and at a global scale is not widely available. Where it has been done, most human cases and outbreaks are caused by either *Cryptosporidium parvum* which has a wide host range including livestock, or *Cryptosporidium hominis* which is human-adapted [9]. People-related exposure, environmental and social risk factors have been identified epidemiologically for *C. hominis* whereas those for *C. parvum* are mainly animal-related [10, 11]. Species identification therefore provides a useful differentiation between human and zoonotic sources [12], and since January 2000 has been undertaken on *Cryptosporidium*-positive stools voluntarily submitted to the national Cryptosporidium Reference Unit (CRU) and, where possible, on positive samples from suspected sources or vehicles of infection, as part of services provided for case and outbreak investigation, management and control in England and Wales [13].

**Fig. 1:**
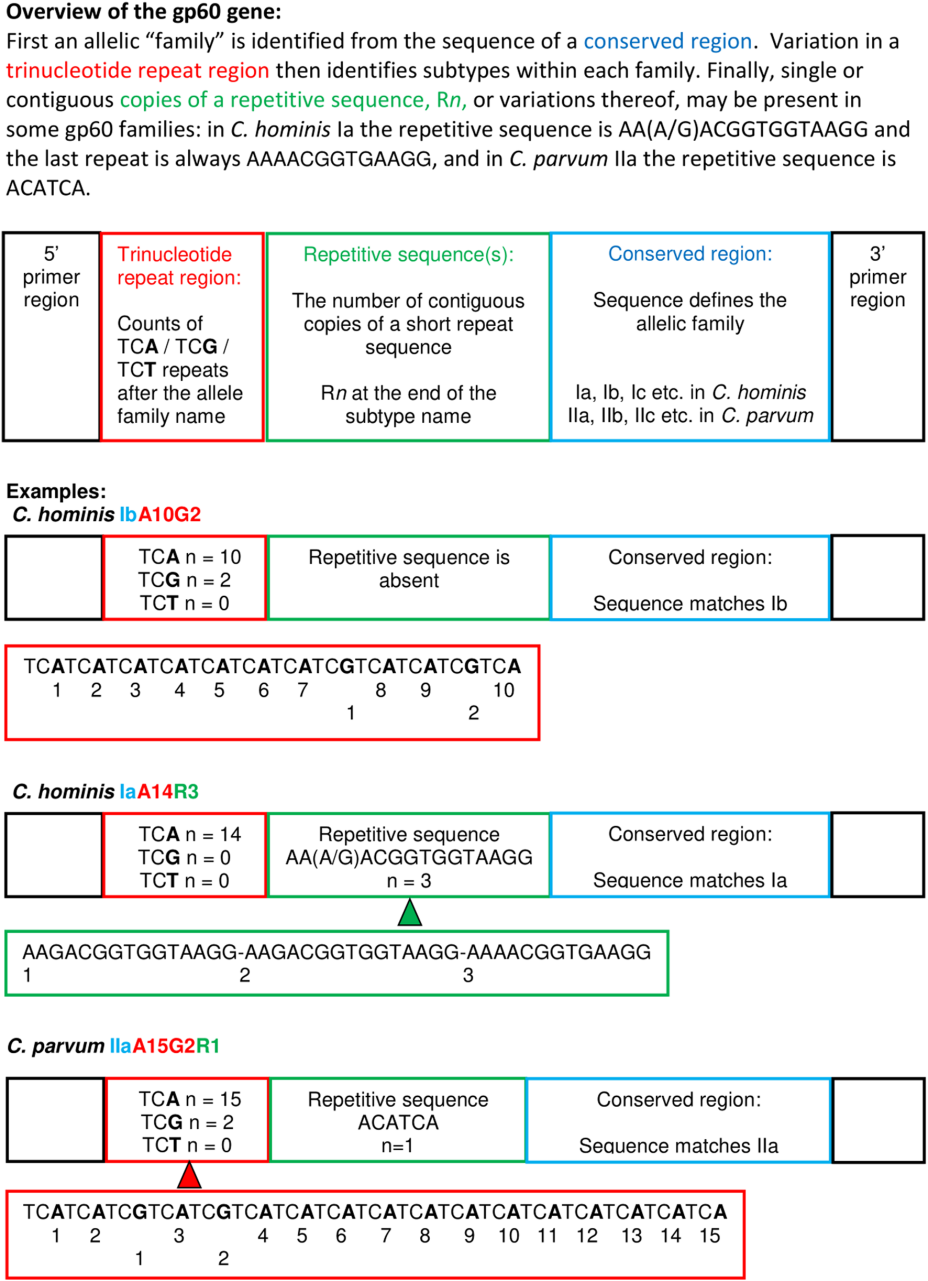
Derivation of *gp60* nomenclature for *Cryptosporidium hominis* and *C. parvum*

Since 2003 further characterisation of *C. parvum* and *C. hominis* has been undertaken at the CRU by sequencing part of the hyper-variable 60 kDa glycoprotein (*gp60*) gene [14]. The nomenclature of *gp60* genotypes has been described in detail [9, 15, 16] and is illustrated in Fig. 1. Where *gp60* genotypes have been investigated in human cryptosporidiosis in Europe, IbA10G2 is the most common among *C. hominis* and IIaA15G2R1 usually, but not always, the most common among *C. parvum* [5]. Identifying the *gp60* genotypes has been an important supplement to epidemiological and environmental data in characterising the infection/contaminants in outbreaks, relating transmission vehicles, and linking cases [9]. Previous analysis of waterborne outbreaks that occurred in England and Wales between January 2001 and December 2010 demonstrated strengthening of the evidence for the association with water provided by identifying the *Cryptosporidium* species and *gp60* subtypes, as well as monitoring the spread of outbreak-associated strains [17]. Likewise, in animal contact-related outbreaks analysed between 1999–2008, animals were linked by *gp60* subtype to human cases in three outbreaks [18]. Here, we provide an updated overview of the *Cryptosporidium* species and *gp60* genotypes associated with outbreaks in England and Wales from January 2009 to December 2017, ahead of the introduction of a validated multilocus genotyping scheme.

## Results

A total of 178 *Cryptosporidium* outbreaks were identified in England and Wales in the years 2009–2017 (Table 1); 123 (69%) had been reported to eFOSS, 43 of which were unique to that dataset. Of the 135 outbreaks in the CRU database, 55 had not been reported to eFOSS. Stools were sent to the CRU for speciation, in 131 outbreaks. Of the 178 outbreaks, 82 (46%) involved recreational waters, 74 (42%) animal contact, 4 (2%) environmental contact or outdoor recreation, 4 (2%) person-to-person spread, 3 (2%) food, 2 (1%) drinking water supplies, and 9 (5%) outbreaks with unknown or various exposures (Table 1). Comparison of the outbreaks unique to each dataset showed that there was no significant difference in the proportion of animal contact outbreaks (*χ*^2^ = 0.63, *P* = 0.43) or recreational water outbreaks (*χ*^2^ = 0.41, *P* = 0.52) (data not shown); therefore, a combined database of all 178 outbreaks was used.

**Table 1:** Outbreak vehicles and *Cryptosporidium* species identified in cases in England and Wales, 2009–2017

Vehicle or source (median; range)^a^	Setting	Number of outbreaks				
		Total	No. genotyped (%)	*C. parvum*	*C. hominis*	Both species
All outbreaks (5; 2–1589 cases)		178	131 (74)	69	60	2
Recreational water (5; 2–70 cases)		82	59 (72)	6	52	1
	Swimming pool	72	55	6	48	1
	Hydrotherapy pool	5	1	0	1	0
	Baby swimming pool	4	2	0	2	0
	Paddling pool (outdoor)	1	1	0	1	0
Animal contact (5; 3–41 cases)		74	53 (72)	53	0	0
	Open/petting/educational farm	52	38	38	0	0
	Commercial farm	7	4	4	0	0
	College farm events	8	5	5	0	0
	Student animal handling classes	5	4	4	0	0
	Lambs taken to institutions	2	2	1	0	0
Environmental contact (7; 5–14 cases)						
	Environmental contact	4	3 (75)	3	0	0
Person-to-person spread (5; 3–14 cases)						
	Daycare nursery	4	3 (75)	0	3	0
Drinking water		2	2 (100)			
	Mains water supply (23 cases)	1	1	0	1	0
	Private water supply (12 cases)	1	1	1	0	0
Food		3	3 (100)			
	Ready-to-eat salad (300 cases)	1	1	1	0	0
	Milk (6 cases)	1	1	1	0	0
	Sandwiches containing salad; coffee shop (192 cases)	1	1	1	0	0
Not known (20; 4–1589 cases)		9	8 (89)	3	4	1
	Community	4	3	1	1	1
	Not known	3	3	3	0	0
	School	1	1	1	0	0
	Open prison	1	1	1	0	0

^a^Median number of confirmed cases; range, where known

The number of confirmed cases was known for 172 outbreaks, affecting a total of 3854 cases, median 5 (range 2–1589) per outbreak. Of the outbreaks with a known vehicle or source, the food-borne outbreaks had the most cases (Table 1).

**Fig. 2:**
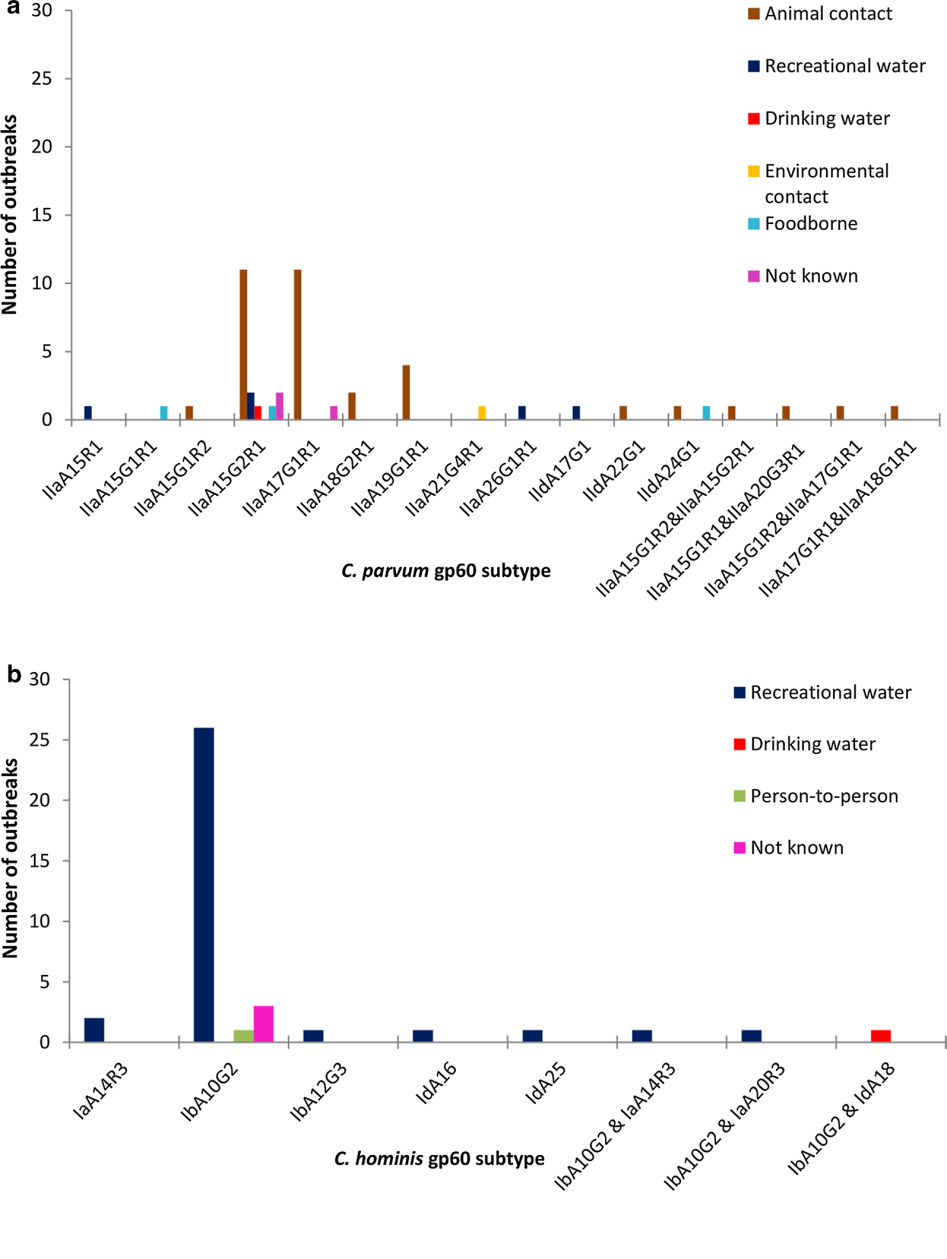
*gp60* subtypes identified in 86 *Cryptosporidium* outbreaks in England and Wales, 2009–2017. **a** Subset of 48 *C. parvum* outbreaks. **b** Subset of 38 *C. hominis* outbreaks

The *Cryptosporidium* species infecting patients were identified in 131 (74%) of outbreaks (Table 1), as samples were not always sent for genotyping. More outbreaks were caused by *C. parvum* (*n* = 69) compared to *C. hominis* (*n* = 60). In two outbreaks, patients infected with either species were identified. *Gp60* subtyping was undertaken in 86 outbreaks. The *C. parvum* outbreaks involved 14 *gp60* subtypes compared to 7 in the *C. hominis* outbreaks (Fig. 2). The itemised list of outbreaks can be viewed in Additional file 1: Table S1.

**Fig. 3:**
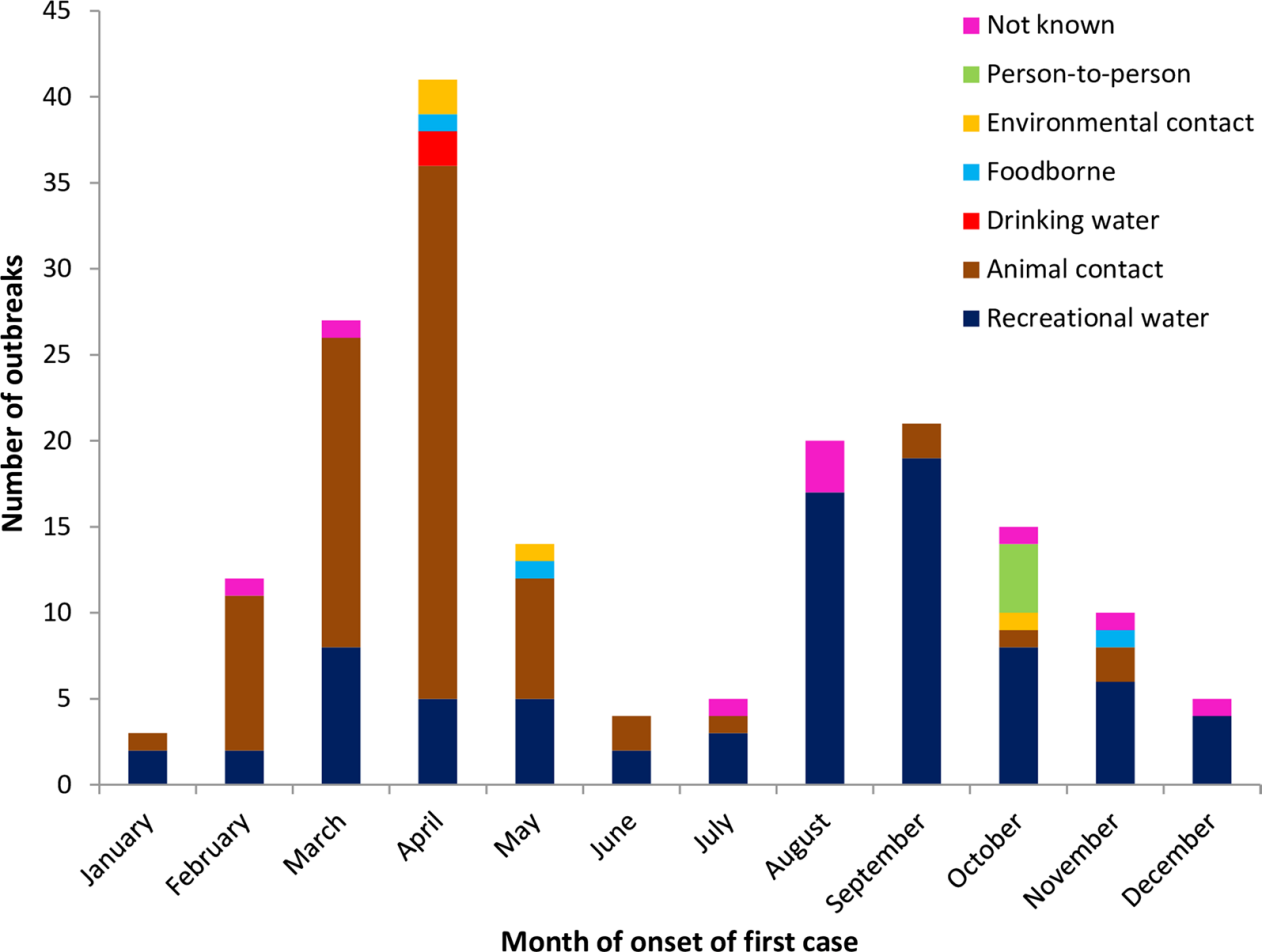
Seasonal distribution of *Cryptosporidium* outbreaks England and Wales 2009–2017

The distribution of outbreaks by vehicle varied seasonally (Fig. 3), with animal contact outbreaks predominating in the first half of the year and recreational water outbreaks mainly in the second half of the year. Outbreaks involving person-to-person spread were all in October, and both drinking water outbreaks were in April.

Of the 74 animal contact outbreaks, the *Cryptosporidium* species infecting patients was identified in 53 (72%); all were *C. parvum*. *Gp60* subtypes were investigated in 35 animal contact outbreaks, and 9 subtypes were identified; IIaA15G2R1 and IIaA17G1R1 were most common (Fig. 2a), and although both are widely distributed globally [9] IIaA17G1R1 has only been reported in one outbreak previously which was also in the UK [18]. Two outbreaks were caused by IIaA18G2R1 and although there are numerous reports in cattle and this subtype was previously linked to calf contact [18], lambs were implicated epidemiologically in both the outbreaks here and were found to be shedding this subtype in one of the outbreak investigations (Table 2). Despite IIaA15G1R1 being the most common subtype in clusters of cases and outbreaks in Scotland [19], it was involved in just two outbreaks in England and Wales, one linked to milk, and the other to animal contact with IIaA20G3R1 which previously caused an outbreak in south-east Ireland linked to a public drinking water supply [20]. In three other outbreaks, two subtypes were also identified in different patients (Fig. 2a), indicating that there may have been contact with different animals at the same setting or that animals were co-infected. In four outbreaks, IIaA19G1R1 was identified but in one of these outbreaks all nine case samples had a nonsynonymous substitution in the *gp60* sequence (adenine to guanine transition at nucleotide 191, see GenBank accession number MK391452) which altered the amino acid sequence, changing the aspartic acid residue to a glycine residue. This substitution has been reported previously elsewhere, but the impact upon the resultant glycoprotein is unknown.

**Table 2:** Subset of 25 outbreaks where *Cryptosporidium* was recovered from a suspected source or vehicle

eFOSS reference or outbreak number^a^ [publication]	Region	Year	Month of onset of first case	Setting	No. of cases (lab-confirmed)	*Cryptosporidium* species and *gp60* genotype in cases	*Cryptosporidium* detections in suspected source or vehicle (species and *gp60* genotype where identified)
Recreational water							
2009/64	Wales	2009	August	Swimming pool (leisure pool)	106 (46)	*C. hominis*	Presumptive oocyst detected in filter sand
14	South-East	2009	November	Swimming pool (leisure pool)	15 (11)	*C. hominis*	Oocysts detected in leisure pool, strainer basket, and sand from two filters
2012/78	Wales	2012	August	Swimming pool (leisure pool)	23 (23)	*C. hominis*	Oocysts detected in pool water
2014/25	South-East	2014	March	Swimming pool (leisure pool)	20 (14)	*C. hominis* IbA10G2	Oocysts detected in pool water (*C. hominis* IbA10G2)
2014/123	South-East	2014	September	Swimming pool (leisure pool)	15 (15)	*C. hominis* IaA14R3	Oocysts detected in filter sand and backwash
151	West Midlands	2016	May	Swimming pool (leisure pool)	10 (9)	*C. hominis* IbA10G2	Oocysts detected in pool water (*C. hominis*)
152	South-West	2016	May	Swimming pool (leisure pool)	25 (25)	*C. hominis* IbA10G2	Oocysts detected in pool water and filter sand (*C. hominis* IbA10G2)
2015/50	North-West	2015	July	Swimming pool (club use)	18 (4)	*C. parvum* IIaA15G2R1	Oocyst detected in filter sand
2012/80	South-West	2012	August	Swimming pool (holiday park)	20 (6)	*C. hominis*	Oocysts detected in pool water
2011/118	London	2011	June	Swimming pool (warm, for baby swim lessons)	7 (7)	*C. hominis* IbA10G2	Oocysts detected in pool water, sand from two filters and backwash
2013/86	Yorkshire and the Humber	2013	July	Paddling pool, outdoor	70 (70)	*C. hominis* IbA10G2	Oocysts detected in pool water (*C. hominis* IbA10G2)
Animal contact							
2011/38	Yorkshire and the Humber	2011	May	Open farm, same premises as 2013/44	12 (12)	*C. parvum*	Lambs (*C. parvum*)
2013/44 [24]	Yorkshire and the Humber	2013	April	Open farm, same premises as 2011/38	45 (32)	*C. parvum* IIaA19G1R1	Lambs (*C. parvum* IIaA19G1R1)
2013/31	South-East	2013	March	Open farm	18 (15)	*C. parvum* IIaA15G2R1	Lambs and a donkey (*C. parvum*)
2015/27	South-East	2015	January	Open farm	10 (8)	*C. parvum* IIaA15G1R2; IIaA15G2R1	Goat kid and lambs (*Cryptosporidium* spp.)
2015/31	West Midlands	2015	April	Open farm	30 (14)	*C. parvum* IIaA17G1R1	Lambs (*C. parvum* IIaA17G1R1 and IIaA21G3R1)
2016/29	Yorkshire and the Humber	2016	March	Open farm	54 (33)	*C. parvum* IIaA15G1R2; IIaA17G1R1	Calf (*C. parvum* IIaA17G1R1); piglets (*C. suis*); lambs (*C. ubiquitum*)
2016/19	Yorkshire and the Humber	2016	February	Open farm, same premises as 2017/12	9 (9)	*C. parvum* IIaA18G2R1	Lambs (*C. parvum* IIaA18G2R1 and *C. xiaoi*)
2017/12	Yorkshire and the Humber	2017	April	Open farm, same premises as 2017/12	5 (5)	*C. parvum* IIaA17G1R1	Lambs (*C. parvum* IIaA17G1R1, *C. parvum* and *C. xiaoi*)
170	Wales	2017	April	Open farm	7 (7)	*C. parvum* IIaA15G2R1	Lambs (*C. parvum* IIaA15G2R1)
2012/22	Wales	2012	March	Open farm (community farm)	15 (10)	*C. parvum* IIaA15G2R1	Droppings from co-penned lambs and goats (*C. parvum* IIaA15G2R1)
2009/19	North-West	2009	May	Commercial farm (open day)	155 (41)	*C. parvum* IIaA17G1R1	Calves (*C. parvum* IIaA17G1R1); goats (*C. parvum* IIaA17G1R1 and *C. xiaoi*)
2016/24	Wales	2016	March	Agricultural College farm	24 (17)	*C. parvum* IIaA15G2R1	Sheep and lambs (*C. parvum* IIaA15G2R1 and *C. xiaoi*)
Drinking water							
77	South-West	2013	April	Mains drinking water	23 (23)	*C. hominis* IbA10G2 and IdA18	*C. hominis* IbA10G2, *C. parvum* and *C. andersoni* in source waters; *C. hominis* and a gastric species in treated water
Food-borne							
78	Yorkshire and the Humber	2013	April	Milk from an on-farm dairy, pasteurisation problems	11 (6)	*C. parvum* IIaA15G1R1	Calf (*C. parvum* IIaA15G1R1)

^a^See Additional file 1: Table S1

Most of the other *C. parvum* subtypes found in animal contact outbreaks (Fig. 2a) have been only reported previously in sporadic cases and animals, but food-borne and animal contact outbreaks caused by IIdA24G1 have been reported in Sweden [21, 22].

In 12 (16%) animal contact outbreaks, *C. parvum* was also confirmed in animals and the same *gp60* subtypes found in patients were identified in nine of these, providing further microbiological evidence for the source of the outbreak (Table 2). Lambs were most commonly implicated as sources of infection. Two farm premises were each linked to two outbreaks each within the study period (Table 2); at one farm the *C. parvum* subtypes differed between the two outbreaks which were 1 year apart, most likely because orphan lambs were brought in from different holdings [23]. Other *Cryptosporidium* species and *gp60* subtypes were also detected in animal samples as part of outbreak investigations, including species considered zoonotic such as *C. ubiquitum* (Table 2).

*Cryptosporidium parvum* was also the exclusive cause of the three food-borne outbreaks. In an outbreak linked by descriptive epidemiology and environmental investigations to under-pasteurised milk from an on-farm dairy, IIaA15G1R1 was detected in patients and a calf at the farm. Analytical epidemiology (case-control studies) linked the other two food-borne outbreaks to consumption of ready-to-eat loose leaf salad in which patients were infected with IIaA15G2R1 [24], and to eating sandwiches containing salad and coffee shops which was associated with infection with a more rare subtype, IIdA24G1 [25].

The *Cryptosporidium* species infecting patients were identified in 59/82 (72%) recreational water outbreaks (Table 1); of these 52/59 (88%) outbreaks were *C. hominis*, six were *C. parvum* and both these species were involved in one outbreak. All of the recreational waters were treated and most were indoor swimming pools; the only outdoor venue was a paddling pool where the outbreak was caused by *C. hominis*. In 31 recreational water outbreaks a single *gp60* subtype was identified, most commonly IbA10G2 (26 outbreaks) (Fig. 2b); this subtype predominates in northern Europe [5] and has been reported in outbreaks previously in the UK [17]. Three swimming pool-related outbreaks involved IaA14R3, which was reported to have caused an outbreak in the US linked to a water park in 2001 [9]. Of the other *C. hominis* subtypes detected, IaA20R3 caused an outbreak in the USA in 2008 [9] but IbA12G3, IdA16, and IdA25 have not been reported in outbreaks elsewhere. Subtype IIaA26G1R1 was a new finding with no previous reports. In two recreational water outbreaks two *C. hominis* subtypes were identified in different patients (Fig. 2b), indicating multiple contamination events.

Four *gp60* subtypes were identified in the five of the six *C. parvum* outbreaks linked to recreational waters. Two outbreaks were caused by the common IIaA15G2R1, and three involved subtypes that were not found in animal contact outbreaks; IIaA15R1 is a rarely reported subtype, IIdA17G1 caused a food-borne outbreak in Finland [26] and IIaA26G1R1 has not been reported previously.

*Cryptosporidium* is not a routine test parameter for recreational waters and sampling and testing can be difficult to arrange, is expensive and not always warranted [27]. Nevertheless, *Cryptosporidium* oocysts were detected in samples from 11 (13%) outbreak premises. Not all were submitted for genotyping, but in four outbreaks the oocysts were confirmed as *C. hominis*, of which three were subtyped and found to be IbA10G2. No other *Cryptosporidium* species or subtypes were found.

One of the two drinking water outbreaks involved a mains water supply, and consumers became infected with *C. hominis* IbA10G2 and IdA18; *C. hominis* was confirmed in the water supply but *gp60* sequences were not obtained. Both subtypes have been implicated in drinking water-related outbreaks previously [9, 28]. The other drinking water outbreak was caused by *C. parvum* IIaA15G2R1 and was related to holiday cottages on a private water supply which was not sampled for *Cryptosporidium*.

Three of the four outbreaks linked to person-person spread were caused by *C. hominis* and one was not genotyped. In one outbreak, IbA10G2 was confirmed in patients. Three of the four environmental contact-related outbreaks were caused by *C. parvum* and one was not genotyped. Samples from only one were subtyped, with a rare subtype, IIaA21G4R1, identified among a group of soldiers after a military exercise in a rural area. Of nine outbreaks with no clear exposure, four were *C. hominis* (three IbA10G2, one not subtyped), three *C. parvum* (two IIaA15G2R1 and one IIaA17G1R1), one involved both species but not subtyped and in the other outbreak, no case samples were sent for typing.

Representative *gp60* sequences from the outbreaks described here have been deposited into GenBank under accession numbers MK391438–MK391457, KF287126 and KT634306.

## Discussion

We analysed a combined dataset of 178 outbreaks from voluntary notifications to eFOSS and those that came to the attention of the CRU during outbreak investigations. In England and Wales, there is no legal obligation to report outbreaks apart from those that are considered food-borne [29]. All surveillance systems are subject to underreporting, and eFOSS is no exception as it relies upon the voluntary participation of a wide range of professional groups and organisations for it to function effectively. National surveillance systems should therefore be streamlined as far as possible to make it easier for lead investigators to notify and report conclusions of their outbreak investigations, and the effectiveness of these systems should be reviewed on a regular basis. Once an outbreak has been identified and PHE alerted, the rate of return of eFOSS forms has been reported to be 80% [30], so further encouragement of lead investigators to notify the outbreak initially would lead to an improvement in reporting.

The most common vehicle of *Cryptosporidium* outbreaks was recreational water, especially swimming pools. This concurs with previous findings, and *Cryptosporidium* is the predominant aetiology of infectious disease linked to treated recreational water venues in England and Wales [31] and the USA [32]. There are difficulties and inadequacies in preventing contamination and treating pool water to disinfect and remove this small, chlorine-resistant parasite [33]. The Pool Water Treatment Advisory Group has published guideline standards for swimming pools [34, 35], and previous analysis of swimming pool-related outbreaks in England and Wales identified failures across the operation and management of pools [36]. Although there is no requirement for compliance assessment for swimming pools in the UK, a check list to identify failures as part of the acute response to outbreaks is available [34]. Systematic route cause analysis of outbreaks could direct efforts for further improvement.

Where identified, outbreaks were caused by *C. parvum* or *C. hominis.* The only other species reported to have caused an outbreak in England and Wales was *Cryptosporidium cuniculus* which caused a mains drinking water outbreak in 2008 [37]. Encouraging all laboratories to send *Cryptosporidium*-positive stools for identification and subtyping would ensure more outbreaks were characterised, and may assist in outbreak identification through more sensitive exceedance monitoring. The IIdA24G1 outbreak was large, widespread, and linked epidemiologically to food-borne transmission. It was identified initially through surveillance activities, and the epidemiology refined by identification of this unusual *gp60* subtype, which helped identify cases from background and put a time frame on the outbreak. Exceedance monitoring may be improved by more extensive application and inclusion of species and subtyping data in routine surveillance data capture and this is currently in development.

The seasonal distribution of outbreaks was remarkably similar to that of sporadic cases, with *C. parvum* being most prevalent in the spring and *C. hominis* in the autumn [13], indicating there may be unrecognised outbreaks, and a burden of sporadic illness, linked to similar seasonal exposures. Although there were only four outbreaks attributed to person-to-person spread, usually in child daycare centres, all were in October and the effect of mixing children together after the summer holidays should be investigated further as a driver for seasonal increase in sporadic cases.

The preponderance of swimming pool-related outbreaks caused by *C. hominis*, and animal-contact related outbreaks caused exclusively by *C. parvum*, concurs with earlier reports [17, 18] and reflects the source of oocysts in each. Despite occasional reports of *C. hominis* infections in animals, their role in human infection is currently unclear [38]. There seems to be little evidence from epidemiological studies for animal involvement in human transmission of this species in England and Wales [10, 11], and *C. hominis* was not found when sampling animals at premises associated with human outbreaks in this study. Zoonotic species other than *C. parvum*, such as *C. ubiquitum*, were found in animals but were not identified in outbreak-related cases.

Although few in number, the largest outbreaks were food-borne, and highlights the emergence of food, especially ready-to-eat salad leaves, as a vehicle. That food-borne outbreaks were caused by *C. parvum* is indicative of animal sources, most likely during production. Unlike drinking water, where implementation of improved catchment and source water protection, monitoring, and water treatment has reduced the number and size of water-borne *Cryptosporidium* outbreaks [39], fewer controls have been implemented in the food chain [40]. Likewise, there is a need to further control contamination and dispersal of *Cryptosporidium* through swimming pools, where not only the number of outbreaks but also the finding of unusual genotypes illustrates their potential for transmission. In the USA, the rapid emergence and spread of a virulent subtype (IaA28R4) in 2008 was linked to dispersal through swimming pools [41]. Although now more common than IbA10G2 in the USA, this subtype has yet to emerge in England and Wales.

The “hypertransmissible” subtype IIaA15G2R1 that caused most *C. parvum* outbreaks has been reported commonly in sporadic and outbreak cases and in a wide range of livestock, wild and other animals especially cattle [38]. There is much emphasis on sampling cattle as a host for *C. parvum* but small ruminants such as lambs can also be an important zoonotic source in some settings, especially open/petting farms [42]. However, genetic subpopulations overlap between IIaA15G2R1 and other *gp60* subtypes [16, 38], and a multilocus genotyping scheme would provide further molecular epidemiological refinement [43]. Undoubtedly other *gp60* subtypes cause human infection and outbreaks, but their lack of detection is most likely due to the lack of both case and outbreak surveillance globally. If all samples were subtyped (especially using a mutlilocus scheme) we would probably see greater diversity, and detect more outbreaks.

The proportion of outbreaks where suspected source material that was investigated to identify the *Cryptosporidium* spp. in recreational water and animal contact outbreaks (13% and 16% respectively) was comparable to that reported for food-borne outbreaks caused by other pathogens where microbiological results were reported as providing evidence supporting the conclusions of the outbreak control team [44, 45]. However, food items were not tested in the outbreaks reported here. Sampling and testing food such as ready-to-eat salad leaves is challenging: retrieving appropriate samples may be impossible as by the time the outbreak is identified none of the food remains for testing, and although there is an ISO standard for testing leafy greens for *Cryptosporidium* [8], we are not aware of any laboratories in the UK that hold accreditation for this test. Although there is no standard method for testing milk, sampling the calves on the farm where the implicated milk was produced and processed provided a microbiological link between the cases and the herd. Sampling source animals has greater potential to yield pathogens such as *Cryptosporidium* than sampling foods or the environment.

The public health benefit of identifying infecting *Cryptosporidium* species and subtypes in outbreak-associated cases is two-fold: (i) identifying and strengthening epidemiologic links between cases; and (ii) indicating possible exposures and outbreak sources. If meaningful samples are available from the latter then there are added benefits, but especially for food these are rarely available and standard methods for detection are lacking or not implemented. Linking cases with each other, refining epidemiology is especially useful in outbreaks identified by exceedence monitoring where clear epidemiological links do not readily emerge from existing data. To better understand the epidemiology of *Cryptosporidium*, molecular characterisation of *Cryptosporidium* specimens needs to shift from predominantly supporting outbreak investigations to becoming nationally systematic.

## Conclusions

The degree to which *Cryptosporidium* outbreaks are unreported is not known but identified trends may reflect the primary vehicles or settings of transmission. Improved outbreak reporting needs to be enabled, and route cause analysis used to identify measures for reductions in exposure. Characterisation of *Cryptosporidium* spp. and subtypes needs to shift from predominantly supporting outbreak investigations to becoming nationally systematic, enabling more sensitive and specific exceedence monitoring and identification of large scale outbreaks that may not be geographically defined. This is an emerging trend and has been seen with food-borne outbreaks. More discriminatory, multilocus subtyping should be implemented to investigate cases and outbreaks.

## Methods

The aim was to describe and analyse trends in the *Cryptosporidium* species and *gp60* genotypes identified in human outbreaks of cryptosporidiosis in England and Wales from January 2009 to December 2017. Definitions used to define an outbreak were: an incident in which two or more people experienced a similar illness and linked in time or place, or a greater than expected rate of *Cryptosporidium* reports compared with the usual background rate for a place and time. *Cryptosporidium* outbreaks were extracted from the eFOSS database and from records for those that also came to the attention of the national CRU during outbreak investigations. The proportions of outbreak routes of transmission were compared between the two databases by uncorrected Chi square and a *P*-value of 0.05 was regarded as significant. The databases were reconciled by PHE centre, setting/place name, postcode, dates of first and last known cases, and populated with *Cryptosporidium* species and *gp60* subtypes identified in the stools of cases and any additional samples tested. The outbreaks were analysed for trends in vehicles and settings, season, and associated *Cryptosporidium* species and *gp60* subtypes. The CRU archive and the NCBI nucleotide DB and PubMed were searched for previous reports of subtypes found.

To identify species, *Cryptosporidium* positive stools were sent by primary diagnostic laboratories to the national CRU, generally within 5 days of collection [13]. Oocysts were separated from faecal material by salt flotation, disrupted by boiling, and DNA extracted using proteinase K digestion and a spin column kit (QIAamp DNA mini kit, Qiagen, Hilden, Germany) as described previously [13]. Samples were screened for *C. parvum* and *C. hominis* using a duplex real-time PCR assay [46] and other species were sought using a nested PCR targeting the *SSU* rDNA gene [47]. A nested PCR targeting the *gp60* gene was used to subtype *C. parvum* and *C. hominis* samples known or suspected to be part of outbreaks as described previously [48]; to simplify workflow a cocktail of single round PCR primers was developed and used from 2015, as described previously [49]. PCR amplicons were subjected to bidirectional sequencing (Applied Biosystems 3500XL) and sequence similarities searched for in the NCBI Blastn website tools. *Gp60* subtypes were confirmed by manual identification of trinucleotide repeats and other repeat sequences (Fig. 1). The findings were contextualised at the time to inform outbreak investigations and updated for this article.

In animal contact outbreaks, animals were sampled by a Veterinary Investigation Officer if requested by the outbreak control team and tested using immunofluorescence microscopy (Crypto-cel, Cellabs) at the Animal and Plant Health Agency’s central laboratory, Weybridge. *Cryptosporidium*-positive samples were sent to the CRU for genotyping as described above. In recreational and drinking water outbreaks, sampling and testing was undertaken as described in [7] if requested by the outbreak control team. *Cryptosporidium* positive microscope slides sent to the CRU for genotyping were processed as described previously [37] until 2015. After 2015 DNA extraction from slides was done using a chelex-based method as described previously [50].

## Additional file

**Additional file 1: Table S1.** A database of 178 *Cryptosporidium* outbreaks in England and Wales, 2009–2017.

## Abbreviations

CRU: Cryptosporidium Reference Unit
eFOSS: Outbreak Electronic Foodborne and Non-Foodborne Gastrointestinal Outbreak Surveillance System
gp60: 60 kDa glycoprotein
PHE: Public Health England
